# Antibacterial Action of Jineol Isolated from *Scolopendra subspinipes mutilans* against Selected Foodborne Pathogens

**DOI:** 10.3389/fmicb.2017.00552

**Published:** 2017-03-28

**Authors:** Vivek K. Bajpai, Shruti Shukla, Woon K. Paek, Jeongheui Lim, Pradeep Kumar, MinKyun Na

**Affiliations:** ^1^Microbiome Laboratory, Department of Applied Microbiology and Biotechnology, Yeungnam UniversityGyeongsan, South Korea; ^2^Department of Energy and Materials Engineering, Dongguk UniversitySeoul, South Korea; ^3^National Science Museum, Ministry of Science, ICT and Future PlanningDaejeon, South Korea; ^4^Department of Forestry, North Eastern Regional Institute of Science and Technology (Deemed University)Nirjuli, India; ^5^College of Pharmacy, Chungnam National UniversityDaejeon, South Korea

**Keywords:** antimicrobial effect, jineol, foodbrone pathogens, scanning electron microscopy

## Abstract

This study was undertaken to assess the antibacterial potential of 3,8-dihydroxyquinoline (jineol) isolated from *Scolopendra subspinipes mutilans* against selected foodborne pathogens *Escherichia coli* O157:H7 and *Staphylococcus aureus* KCTC-1621. Jineol at the tested concentration (50 μL; corresponding to 250 μg/disk) exhibited significant antibacterial effects as a diameter of inhibition zones (11.6–13.6 mm), along with minimum inhibitory concentration (MIC) and minimum bactericidal concentration values found in the range of (62.5–125 μg/mL) and (125–250 μg/mL), respectively. Jineol also exhibited significant antibacterial effects as confirmed by the reduction in bacterial cell viabilities, increasing release of potassium (K^+^) ions (650 and 700 mmole/L) and 260 nm materials (optical density: 2.98–3.12) against both the tested pathogens, *E. coli* O157:H7 and *S. aureus* KCTC-1621, respectively. Moreover, changes in the cell wall morphology of *E. coli* O157:H7 and *S. aureus* KCTC-1621 cells treated with jineol at MIC further confirmed its inhibitory potential against the tested pathogens, suggesting its role as an effective antimicrobial to control foodborne pathogens.

## Introduction

Foodborne illnesses caused by foodborne pathogenic bacteria affect a huge number of population world-wide. Development of natural alternative means as safe antimicrobials is essential in combating serious foodborne pathogens which pose significant threat to humans (Alwash et al., 2013). Among several studied foodborne pathogens, to some extent, *Escherichia coli* O157:H7, and *Staphylococcus aureus* are known to be causative agents of foodborne diseases. The development of resistance of foodborne pathogens in the commercial antibiotics and the emergence of new strains are widespread concerns (Alwash et al., 2013).

*Staphylococcus aureus* possess the ability to produce enterotoxins as well as contributes in hospital-acquired diseases and food-poisoning, thus, considered a serious foodborne pathogen among others (Pereira et al., 2009). The occurrence of *S. aureus* toxicity depends on the capability of the strain to survive, multiply under a variety of conditions and produce extracellular toxic compounds. Contaminated food, especially undercooked ground beef, raw milk, soft cheese, raw fruits and vegetables are major sources of *E. coli* O157:H7 associated illness. *E. coli* O157:H7 has the ability to produce Shiga toxin, thereby causes bloody diarrhea and sometimes kidney failure (Du et al., 2008).

Consumers, the food industry and the regulatory authorities are concerned about the safety of food from the contamination by foodborne pathogens (Al-Zorekya and Al-Taher, 2015). In the USA, foodborne pathogens have been reported to be the cause of 75% of the foodborne disease outbreaks, involving 68% of all reported cases of foodborne illness (Scot, 2003). Additionally, in Canada, a worth of approximately 500 million dollars is imposed on the treatment of diseases caused by foodborne pathogens surviving on meat or meat-products (Oussalah et al., 2007). In addition, failures in preservation technologies to control foodborne and food spoilage pathogens have reinforced the suggestion for exploring other effective classes of antimicrobials (Awaisheh and Ibrahim, 2009; Negi, 2012). Furthermore, prevalence of synthetic and chemical additives in food and food products has urged an urgent need of application of natural preservatives to meet the consumer acceptability (Shakiba et al., 2011; Shen et al., 2014).

Previous findings support the fact that the use of chemical or synthetic food preservatives imposes a higher rate of health complications, therefore, food processors are often looking for safe and effective antimicrobials of natural origin for food protection and food preservation purposes (Taguri et al., 2004; Santas et al., 2010; Soylu et al., 2010). Also, development of pathogen resistance to commercially available antimicrobials evidences employment of innovative research strategies to explore safe, and effective antimicrobial treatments (Militello et al., 2011). Hence, the present study was designed to isolate a biologically effective 3,8-dihydroxyquinoline (jineol) from a centipede *Scolopendra subspinipes mutilans*, and to determine its antimicrobial potential against selected foodborne pathogenic bacteria.

## Materials and Methods

### Chemicals and Reagents

The nutrient broth (NB) medium was purchased from Difco Ltd., USA. Highly pure quality reagents and chemicals were employed for the test assays. Test samples of jineol were prepared in 1% dimethyl sulfoxide (DMSO) (Sigma–Aldrich, Germany). For absorbance reading, an enzyme-linked immunosorbent assay (ELISA) (Tecan, Infinite M200, Männedorf, Switzerland) was used.

### Test Foodborne Pathogens

*Staphylococcus aureus* KCTC1621 (Gram+), and *E. coli* O157:H7 (Gram-) foodborne pathogens procured from the Korean Collection for Type Cultures (KCTC, Korea) were used in this study. For the growth and culture of strains, NB was used and cultures were incubated at 37°C, followed by maintenance of strains on nutrient agar (NA) slants at 4°C. To reactivate the cultures, cultures were taken out and a loop-full of colonies were inoculated in the fresh NB medium, and incubated for 24 h and 37^o^C. Further, sub-culturing was maintained in NB medium.

### Insect Material

Dried *Scolopendra subspinipes mutilans* specimens were purchased from the herbal market at Geumsan, South Korea, and identified by in-charge of the department. A voucher specimen (CNU-INS 1408) was deposited at the Pharmacognosy Laboratory of the College of Pharmacy, Chungnam National University (Daejeon, South Korea).

### Extraction, Isolation, and Characterization of Jineol

Jineol was isolated from specimens using a chromatographic approach (Lee et al., 2016). Briefly, the dried ethanol (EtOH) extract (110.0 g) was suspended in water and fractionated successively with ethyl acetate (EtOAc) and then *n*-butanol (BuOH) to yield EtOAc-soluble (60.0 g) and *n*-BuOH-soluble (8.0 g) fractions, and residue (40.0 g). The EtOAc-soluble fraction was subjected to vacuum-liquid chromatography (VLC) using hexane-EtOAc 40:1, 20:1, 10:1, and 4:1; hexane-EtOAc-MeOH 2:1:0.2; CHCl_3_-MeOH 6:1; CHCl_3_-MeOH-H_2_O 3:1:0.1 and then washed with MeOH to yield eight fractions (E1–E8). Fraction E4 (8.0 g), which was obtained by eluting with hexane-EtOAc 4:1 was partitioned with hexane and MeOH to afford jineol (60.0 mg).

Jineol: yellowish amorphous powder; ^1^H NMR (300 MHz, CD_3_OD) δ_H_ 8.46 (1H, d, *J* = 2.4 Hz, H-2), 7.42 (1H, d, *J* = 2.4 Hz, H-4), 7.29 (1H, t, *J* = 8.0 Hz, H-6), 7.14 (1H, d, *J* = 8.0 Hz, H-5), 6.87 (1H, d, *J* = 8.0 Hz, H-7), ^13^C NMR (75 MHz, CD_3_OD) δc 154.2 (C-8), 153.0 (C-3), 142.3 (C-2), 134.8 (C-8a), 131.9 (C-4a), 129.0 (C-6), 117.9 (C-5), 117.2 (C-4), 109.0 (C-7) (Moon et al., 1996).

### Determination of Antibacterial Activity of Jineol

A method of agar diffusion was employed to determine the antibacterial activity of jineol using Luria-Bertani (LB) agar plates (Bajpai and Kang, 2010). To make the desired concentrations (0, 10^1^, 10^2^, 10^3^, 10^4^, 10^5^, 10^6^, 10^7^, 10^8^, and 10^9^ cells/mL) of the tested strains, pathogens were cultured in NB medium following incubation at 37°C for 24 h and serially diluted. Further, a method of the microbial plate-count was employed to determine the viable cell numbers. A 100 μL inoculum containing 10^7^ CFU/mL was poured on dried agar plates and spread uniformly using a bacterial plate spreader followed by drying at room temperature. The compound was dissolved in 5% DMSO, and finally 50 μL jineol solution, corresponding to 250 μg/disk was impregnated on a sterilized filter paper (Whatman No. 1) disk. The same solvent used for dissolving the sample was also tested as a negative control. Further, plates followed the incubation of 37°C for 24 h, and zones of inhibition around the disks were measured to confirm the antibacterial activity of test compound in triplicate measurements.

### Determination of Minimum Inhibitory Concentration (MIC) and Minimum Bactericidal Concentration (MBC) of Jineol

A method of twofold serial dilution was employed to determine the MIC of jineol (Bajpai et al., 2013). At first, jineol was dissolved in the DMSO (5%), followed by incorporation into the NB medium to make a 500 μg/mL solution of jineol. Further, serial dilutions of jineol solution were made in NB to obtain 250, 125, 62.5, 31.25, 15.62, and 7.81 μg/mL concentrations of jineol. A 10 μL standardized suspension (˜10^7^ CFU/mL) of each tested organism was inoculated into each tube. Controls were devoid of sample and contained only bacterial inoculum. No bacterial growth in the lowest concentration (μg/mL) of jineol following macroscopic analysis confirmed the MIC of jineol. The cultures (50 μL each) in which jineol concentrations did not show any visual bacterial growth were spread on NA plates in triplicates following incubation of 37°C for 24 h. Finally, the lowest concentration which completely inhibited the formation of CFU on NA plate was referred as MBC of jineol.

### Determination of the Effect of Jineol on Bacterial Viabilities

Freshly grown bacterial colonies of the selected pathogenic bacteria were inoculated in NB and incubated at 37°C for 24 h, and then bacterial cultures were serially diluted to 10^7^ CFU/mL (Shin et al., 2007). To determine the effect of jineol on cell viabilities, each of the tubes containing the bacterial suspension (10 μL; ∼10^7^ CFU/mL) of *S. aureus* KCTC1621 and *E. coli* O157:H7 was inoculated with 100 μL of jineol at its MIC in 890 μL NB broth at 37°C. A time interval of 0, 40, 80, 120, 160, and 200 min was followed to take the sample for counting cell viabilities on NA plates (Bajpai et al., 2013). Counting of CFU was performed after 24 h of incubation at 37°C. Controls were prepared in a similar manner except the treatment of jineol in triplicate.

### Determination of the Effect of Jineol on the Release of Potassium (K^+^) Ions

A previously developed method was adopted for the determination of the effect of jineol on K^+^ ion efflux from the cells of tested bacteria (Bajpai et al., 2013). The concentration of free K^+^ ions from the cell suspensions of *S. aureus* KCTC1621 and *E. coli* O157:H7 was determined for 0, 30, 60, 90, and 120 min following the jineol exposure to bacterial cells at MIC employing sterilized peptone-water. Photometeric measurement of extracellular K^+^ ion was performed at each above-mentioned time interval, using a commercial kit for Calcium/Potassium detection. Controls were also tested in a similar way without the addition of jineol. Data were presented as the release of extracellular K^+^ ion concentration (mmol/L) in a triplicate set.

### Determination of the Effect of Jineol on the Release of 260-nm Absorbing Cellular Materials

The measurements of the release of 260-nm-absorbing components (DNA/RNA) from *S. aureus* KCTC1621 and *E. coli* O157:H7 cells were performed in a 2 mL aliquot of the bacterial inoculum in a sterilized peptone-water. The loss of 260 nm cellular materials is considered a good indication of the antimicrobial efficacy of any test compound. The reaction solution was added of MIC of jineol following incubation at 37°C. Subsequently, cultures treated at 0, 30, and 60 min were collected separately following centrifugation (3,500 × *g*, 10 min) to obtain the cell free supernatants and read for absorbance at 260-nm using an ELISA (Bajpai et al., 2013). Controls tested devoid of jineol. Data were collected at each time point and presented as optical densities (ODs) of the samples.

### Determination of the Effect of Jineol on the Cell Wall Morphology of Foodborne Pathogens

Scanning electron microscopic (SEM) study was executed according to Kim et al. (2007) to examine the effects of jineol on the morphological changes in the cell wall of the selected pathogens, *S. aureus* KCTC1621 and *E. coli* O157:H7 at MIC. Control samples were prepared without jineol. Microscopic examination was performed using a S-4300 SEM Analyzer (Hitachi, Japan).

### Statistical Analysis

Experiments were carried out in a set of triplicate, and the data obtained were presented as mean ± SD following one-way ANOVA statistical analysis coupled with Duncan’s multiple test.

## Results

### Identification and Characterization of Jineol

**FIGURE 1:**
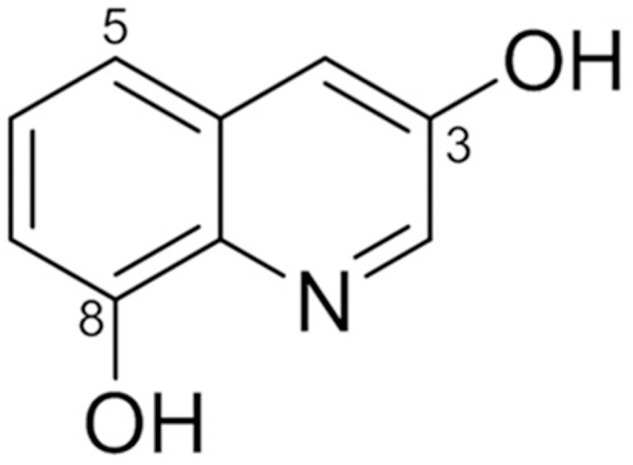
**Chemical structure of jineol isolated from *Scolopendra subspinipes mutilans***.

^1^H NMR data showed signals for five aromatic protons at δ_H_ 8.46 (1H, d, *J* = 2.4 Hz, H-2), 7.42 (1H, d, *J* = 2.4 Hz, H-4), 7.29 (1H, t, *J* = 8.0 Hz, H-6), 7.14 (1H, d, *J* = 8.0 Hz, H-5), and 6.87 (1H, d, *J* = 8.0 Hz, H-7). Typical proton signals for 3-hydroxy quinoline alkaloid were observed at 8.46 (1H, d, *J* = 2.4 Hz, H-2) and 7.42 (1H, d, *J* = 2.4 Hz, H-4). Inspection of the ^13^C NMR spectra revealed nine aromatic carbon signals. Based on its NMR spectroscopic data analyses, the compound was identified as 3,8-dihydroxyquinoline (jineol) (Moon et al., 1996) (**Figure 1**).

### Antibacterial Activity

This study showed antibacterial effects of the test compound jineol as confirmed by the presence of inhibitory zones in agar plates against the tested foodborne pathogenic bacteria, *S. aureus* KCTC1621 and *E. coli* O157:H7. In this assay, jineol exhibited a significant inhibitory effect against both the employed foodborne pathogenic bacteria. The inhibitory effect of jineol in agar plates was confirmed through the diameters of zones of inhibition, which were found to be 11.6–13.6 mm (**Table 1**). It was observed that jineol exhibited antibacterial effects against both the tested bacterial isolates.

**Table 1:** Antibacterial activity of jineol against foodborne pathogens *Staphylococcus aureus* KCTC1621 and *Escherichia coli* O157:H7.

Pathogens	Jineol		
	Zones of inhibition^x^	Susceptibility	
		MIC^y^	^z^MBC
*Staphylococcus aureus* KCTC1621	13.6 ± 0.2^a^	62.5^a^	125^a^
*Escherichia coli* O157:H7	11.6 ± 0.3^b^	125^b^	250^b^

*^x^Diameters of inhibition zones in millimeter; ^y^Minimum inhibitory concentration (values in μg/mL); ^z^Minimum bactericidal concentration (values in μg/mL). Values in the same column with different superscripts are significantly different according to Duncan’s Multiple Range Test (*P* < 0.05). All values were expressed as mean ± SD of three parallel measurements (*n* = 3). Zones of inhibition around the disk were measured in millimeter (mm) using a Vernier’s caliper.*

### MIC and MBC

This assay revealed different susceptibilities of test compound jineol against the tested foodborne pathogens as confirmed by the low and variable MIC and MBC values. As a result, the MIC and MBC values of jineol against the tested pathogens were ranged 62.5–125, and 125–250 μg/mL, respectively (**Table 1**). In this assay, it was observed that jineol had inhibitory effects against both Gram-positive and Gram-negative bacteria.

### Effect on Bacterial Cell Viability

**FIGURE 2:**
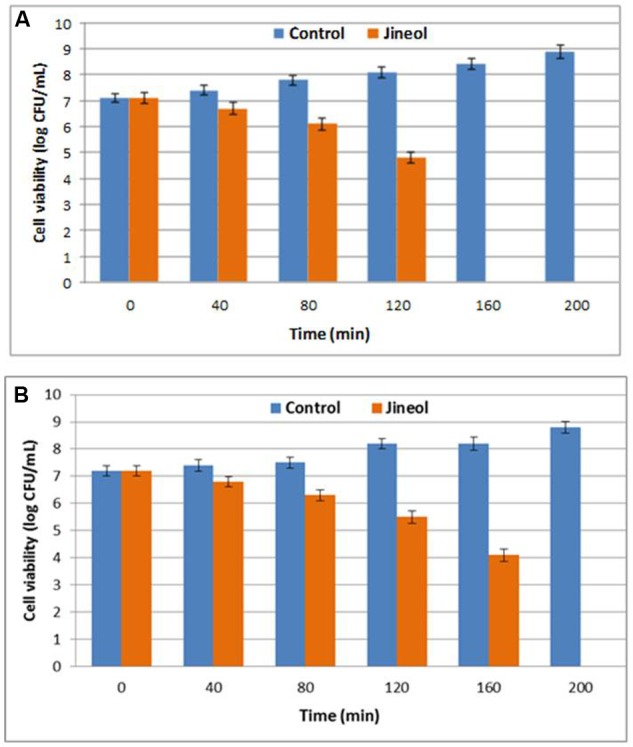
**Effect of jineol on the viability of the tested pathogenic bacteria of *S. aureus* KCTC1621 (A)** and *E. coli* O157:H7 **(B)**. Control without treatment. Data are expressed as mean ± SD (*n* = 3). Values in the same column with different superscripts are significantly different according to Duncan’s Multiple Range Test (*P* < 0.05).

This assay confirmed the antibacterial potential of quinoline alkaloid compound jineol, as confirmed by the reduction in the cell viabilities of the tested pathogens, *S. aureus* KCTC-1621 and *E. coli* O157:H7 when inoculated at MIC (**Figure 2**). Bacterial pathogens when exposed to test compound jineol for 80 min showed no remarkable decrease in the cell viabilities. However, exposure of jineol to pathogen for 160 and 200 min completely inhibited the growth of both the tested pathogens, *S. aureus* KCTC-1621 and *E. coli* O157:H7, respectively (**Figure 2**).

### K^+^ Ion Efflux

**FIGURE 3:**
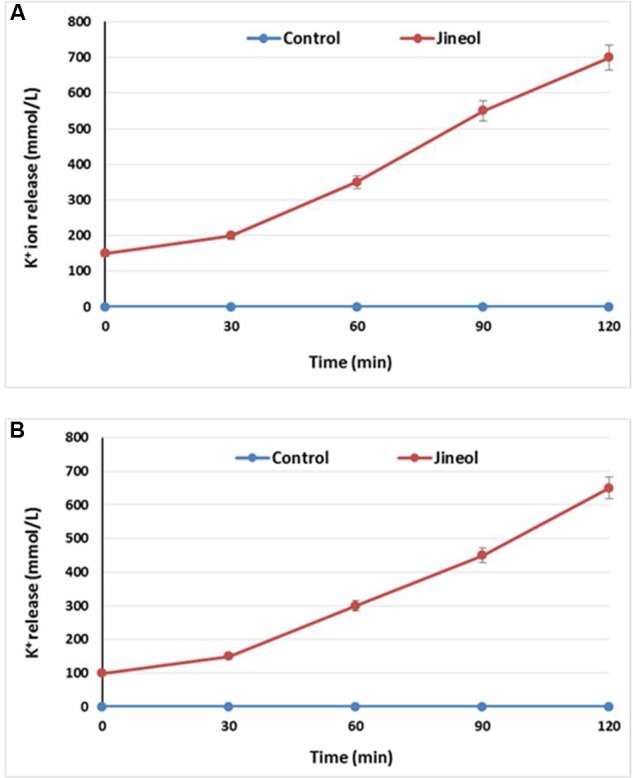
**Effect of jineol on the release rate of extracellular K^+^ ions from *S. aureus* KCTC1621 (A)** and *E. coli* O157:H7 **(B)**. Data are expressed as mean ± SD (*n* = 3). Values in the same column with different superscripts are significantly different according to Duncan’s Multiple Range Test (*P* < 0.05).

Loss of extracellular K^+^ ions from the bacterial cells upon the treatment of specific antimicrobial indicates loss of cell integrity, thus establishing its antimicrobial effect againt the tested bacteria. It was found in this study that jineol at the used concentration exhibited significant antibacterial effect as confirmed by the significant release of K+ ions from the treated bacterial cells of *S. aureus* KCTC-1621 (**Figure 3A**) and *E. coli* O157:H7 (**Figure 3B**). Jineol exhibited time dependent inhibitory effect in this assay. However, no significant release of K+ ions from the control sets were observed.

### Release of 260 nm Absorbing Cellular Materials

**FIGURE 4:**
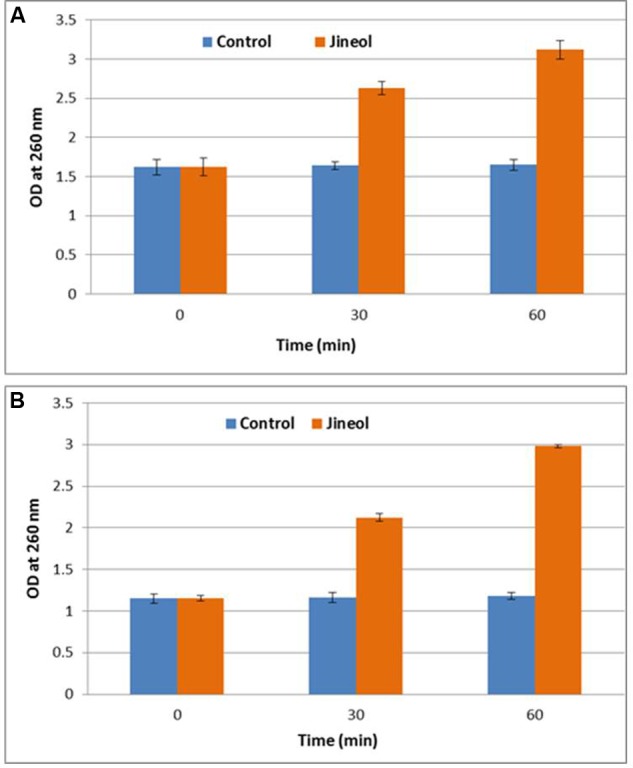
**Effect of jineol on the release rate of 260-nm absorbing material from *S. aureus* KCTC1621 (A)** and *E. coli* O157:H7 **(B)**. Data are expressed as mean ± SD (*n* = 3). Values in the same column with different superscripts are significantly different according to Duncan’s Multiple Range Test (*P* < 0.05).

Since loss of 260 nm cellular materials is considered a good indication of the antimicrobial efficacy of any test compound, this assay confirmed that bacterial cells of tested foodborne pathogens, *S. aureus* KCTC-1621 and *E. coli* O157:H7 upon treatment with jineol at MIC had a severe inhibitory effect in terms of release of 260 nm absorbing materials (DNA and RNA) from them (**Figure 4**). As a result, it was observed that bacterial cells of tested foodborne pathogens, *S. aureus* KCTC-1621 (1.62-3.12) and *E. coli* O157:H7 (1.15-2.98) showed significant differences in their respective ODs as compared to control groups (1.62–1.65) and (1.15–1.18) measured at 260 nm. No significant differences in ODs of control groups were observed in this assay.

### Observation of Morphological Changes in Bacterial Cell Wall

**FIGURE 5:**
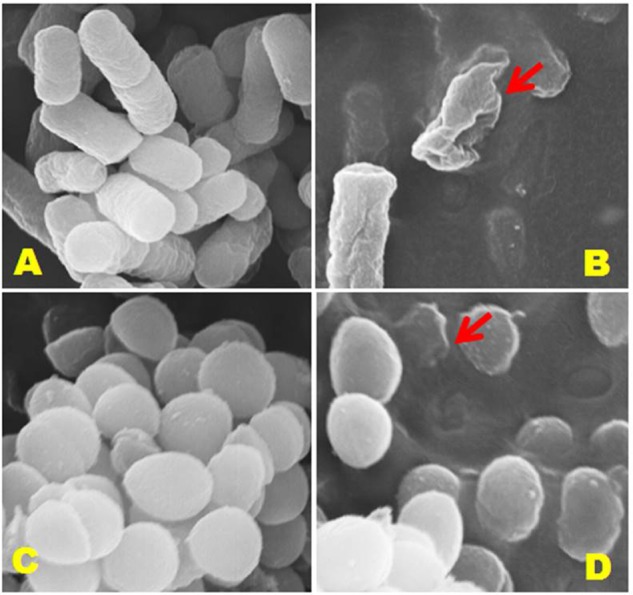
**Scanning electron microscopic (SEM) analysis of *E. coli* 0157:H7 and *S. aureus* KCTC-1621 cells treated with jineol at minimum inhibitory concentration (MIC).** Controls **(A,C)** showing a regular and smooth surface; whereas treated cells **(B,D)** arrows showing disruption and cell lysis, respectively.

It is a vital phenomenon that exposure of the antimicrobial agent to bacterial cells results in the disruption of cell wall, therefore, we performed SEM analysis to further confirm the deteriorating effects of jineol on the cell wall physiologies and morphologies of *S. aureus* KCTC-1621 and *E. coli* O157:H7 cells (**Figure 5**). As a result, jineol significantly altered the cell wall morphology of both the tested pathogens *S. aureus* KCTC-1621 and *E. coli* O157:H7 used at MIC with clear visualization of cell wall damage and cell lysis (**Figures 5B,D**). However, as expected, cells without treatment as a control had intact shape and no morphological changes were observed (**Figures 5A,C**).

## Discussion

The jineol showed significant antibacterial effects against both the tested bacterial isolates. To support the findings our study, recently, a number of quinolines and their derivatives have shown significant ability to inhibit the growth of various pathogenic microbes including foodborne pathogenic bacteria (Cherdtrakulkiat et al., 2016). A number of phytochemicals exhibit higher amount of inhibitory effect against a wide range of pathogenic microbes especially Gram-positive bacteria. Similarly, in the present study, jineol exhibited higher inhibitory effects against *S. aureus* KCTC1621, a Gram-positive bacterium than that of a Gram-negative bacterium, *E. coli* O157:H7. This can be encountered by the phenomenon that the hydrophilic thick cell wall of Gram-negative bacteria made by lipopolysaccharide has ability to block and avoid the accumulation of jinol in the target cell membrane than the single membrane, cell wall structure of Gram-positive bacteria, which might be more permeable to the jineol (Bezic et al., 2003).

The jineol shows the variable MIC and MBC value against the both the foodborne pathogens and the similar reports have confirmed antibacterial efficacy of quinoline alkaloid derivatives in MIC assay, which found to elicit significant antibacterial effects against various foodborne pathogenic bacteria with varied MIC and MBC values (Sibi et al., 2014). Also, isoquinoline alkaloids isolated from the rhizome *Coptis chinensis* were found to exhibit a remarkable inhibitory effect in MIC assay against various pathogens, including *S. aureus* and *E. coli* (Kim et al., 2004).

The exposure of jineol to pathogen for 160 and 200 min completely inhibited the growth of both the tested pathogens. Previous reports have confirmed the inhibitory effects of various phytochemicals isolated from different sources on the cell viabilities of foodborne pathogenic bacteria (Rastogi et al., 2008; Bajpai and Kang, 2010).

The significant release of K+ ions from the jineol treated bacterial cells and no release of K^+^ ions from the control sets were observed. Similar findings were observed when Patra et al. (2015) tested the efficacy of bio-oil on the release of extracellular K^+^ ions from *Bacillus cereus* and *Listeria monocytogenes* cells. Permeability of essential ions such as K^+^ ions is regulated by the bacterial plasma membrane where membrane chemo-structural composition plays a significant role on the release of such ions, and extensive release of these ions from the target bacterial cells could be considered as a disruptive effect of treatment agent on the plasma membrane, hence, validating the antimicrobial role of jineol, as also reported previously (Cox et al., 2001).

This study validated that the increasing release of 260-nm absorbing cellular materials from the target bacterial cells mediated by jineol had a huge influence on the release of cellular metabolites indicating structural damage to the plasma membrane, thereby causing cell death. Such findings on specific antimicrobials have also been confirmed previously (Farag et al., 1989). The results clearly indicate that regulation of nucleic acid or cellular materials from the tested pathogens could be considered a significant indication of membrane structural damage. Similar to our findings, Souza et al. (2013) tested the antibacterial efficacy of essential oil components, carvacrol and thymol against foodborne pathogens, and it was noted that both the compounds were able to cause membrane damaging effects, and eventually caused efflux of 260-nm nuclear components.

Using SEM analysis, it was confirmed that upon treatment with jineol, morphology of treated bacterial cells was dramatically changed or hampered. Similar reports on morphological deteriorating effects of several phytochemicals have been observed previously against various foodborne pathogens (Soylu et al., 2007; Alwash et al., 2013). Such morphological and physiological changes could be caused by the changes in the integrity and fluidity of plasma membrane as well as changes in the composition of the lipid profile of plasma membrane directly hampered by the antimicrobial action of treating agent (Sikkema et al., 1994).

## Conclusion

This study reports isolation of a quinoline compound jineol from *Scolopendra subspinipes mutilans* which was enough able to exhibit significant inhibitory effects against two selected foodborne pathogens. The findings of this study suggest that the antimicrobial action of jineol could be mediated through its efficacy to alter cell membrane permeability parameters of the tested pathogens as demonstrated by the excessive release of K^+^ ions and 260-nm absorbing materials, thus causing membrane disruption as also confirmed during SEM analysis. We conclude that jineol, exhibiting significant antibacterial activity against selected foodborne pathogens could be potential efficacy for using in food and pharma industries as an alternative means of antimicrobial.

## Author Contributions

Author VB and SS designed performed the experiments and write the manuscript. WP assist during experimental work. PK helps in manuscript preparation and editing. JL and MN help in editing and finalizing the manuscript.

## Conflict of Interest Statement

The authors declare that the research was conducted in the absence of any commercial or financial relationships that could be construed as a potential conflict of interest.
